# SUPPRESOR OF GAMMA RESPONSE 1 promotes early onset of endoreplication upon DNA double-strand breaks by inducing *CCS52A1* expression in Arabidopsis roots

**DOI:** 10.1007/s10265-025-01630-y

**Published:** 2025-04-07

**Authors:** Toshiki Wada, Ayako N. Sakamoto, Masaaki Umeda, Naoki Takahashi

**Affiliations:** 1 Department of Life Sciences, School of Agriculture, Meiji University, Kawasaki, Kanagawa 214-8571 Japan; 2 Department of Quantum-Applied Biosciences, National Institutes for Quantum Science and Technology, Takasaki, Gumma 370-1292 Japan; 3 Graduate School of Science and Technology, Nara Institute of Science and Technology, Takayama 8916-5, Ikoma, Nara 630-0192 Japan

**Keywords:** Arabidopsis, CELL CYCLE SWITCH 52 A1, DNA damage, Endoreplication, Root, SUPPRESSOR OF GAMMA RESPONSE 1

## Abstract

Living organisms are constantly at the risk of DNA damage caused by factors such as DNA replication errors, reactive oxygen species, and UV radiation. In plants, DNA damage activates the NAC-type transcription factor SUPPRESSOR OF GAMMA RESPONSE 1 (SOG1) that governs DNA damage responses such as cell cycle arrest, stem cell death, and early onset of endoreplication. However, molecular mechanisms underlying the early induction of endoreplication onset in response to DNA damage remain elusive. In this study, we show that CELL CYCLE SWITCH 52 A1 (CCS52A1), an activator of the APC/C E3 ligase, plays a major role in the early onset of endoreplication in response to DNA double-strand breaks (DSBs) in Arabidopsis roots. Upon DSBs, SOG1 directly binds to the *CCS52A1* locus, thereby inducing its expression. Moreover, early transition to endoreplication in response to DSBs is suppressed in *ccs52a1* knockout roots. Our data propose that increased polyploidy may function as a unique adaptative mechanism to genotoxic stress in plants.

**Supplementary Information:**

The online version contains supplementary material available at 10.1007/s10265-025-01630-y.

## Introduction

DNA is the fundamental molecule, which stores genetic information in cells; therefore, maintaining its integrity is essential for the survival of all living organisms. However, DNA is constantly subjected to damage from both endogenous and exogenous factors. Abiotic stresses, including reactive oxygen species (ROS), UV radiation, ionizing radiation, and excessive levels of aluminum or boron, are the major causes of DNA damage (Cooke et al. 2003; Cruz de Carvalho 2008; Medina et al. 2021; Rounds and Larsen 2008; Sakamoto et al. 2011). Additionally, DNA replication errors and replication fork stalling contribute to genomic instability (Eichman 2023). In contrast to animals, plants are sessile and cannot physically evade unfavorable environmental conditions. Consequently, plants have evolved unique DNA damage response (DDR) systems for maintaining genome integrity.

Upon DNA damage, two evolutionarily conserved serine/threonine protein kinases, ATAXIA TELANGIECTASIA MUTATED (ATM) and ATM AND RAD3-RELATED (ATR), are activated. ATM primarily responds to DNA double-strand breaks (DSBs), a highly deleterious type of DNA damage, while ATR is activated in response to replication stress and the presence of single-stranded DNA (Culligan et al. 2004; Garcia et al. 2003). ATM and ATR phosphorylate and activate SUPPRESSOR OF GAMMA RESPONSE 1 (SOG1), a plant-specific NAC-type transcription factor that is functionally analogous to the p53 tumor suppressor in animals (Sjogren et al. 2015; Yoshiyama et al. 2009, 2013). Activated SOG1 binds to the consensus sequence CTT[N]_7_AAG on target genes to induce their expression and control DNA repair and recombination, cell cycle arrest, and cell death (Bourbousse et al. 2018; Ogita et al. 2018).

One of the primary responses to DNA damage is the activation of cell cycle checkpoints, which temporarily halt cell division to provide sufficient time for DNA repair (Chen et al. 2017). Cell cycle arrest occurs mainly through the suppression of cyclin-dependent kinase (CDK) activity, which is crucial for cell cycle progression by enabling the phosphorylation of specific target proteins in complex with their regulatory cyclins. In response to DNA damage, SOG1 upregulates the expression of several CDK inhibitors, such as SIAMESE-RELATEDs (SMRs) (Yi et al. 2014), which directly bind to CDK–cyclin complexes and suppress their activity. A concurrent reduction in cyclin levels further decreases CDK activity, reinforcing the inhibition of cell cycle progression (Adachi et al. 2011). When DNA damage is irreparable, plants shift their strategy and initiate programmed cell death to eliminate defective cells, thereby preventing the propagation of genomic instability to daughter cells (Fulcher and Sablowski 2009). This process is critical particularly for maintaining the integrity of stem cells, which play a fundamental role in preserving the function of stem cell niche. Together, these responses ensure that plants can effectively manage DNA damage while safeguarding their overall genomic stability.

In Arabidopsis roots, cells actively divide and proliferate in the meristematic zone (MZ) at the root tip. After several rounds of cell division, cells stop dividing and begin endoreplication, a process in which DNA replication is repeated without mitosis or cytokinesis (Traas et al. 1998). Endoreplicating cells increase their nuclear DNA contents in the transition zone (TZ), and eventually initiate rapid cell elongation. Previous studies have shown that DSBs inhibit cell division in the MZ and promote the early onset of endoreplication in the TZ (Adachi et al. 2011). Polyploid cells typically display enhanced metabolic capacity and increased stress resilience, thereby enabling plants to maintain cellular function and viability even under genotoxic conditions (del Pozo and Ramirez-Parra 2015). Therefore, endoreplication, which results in polyploidy, plays a critical role in plant DDR, thereby helping balanced growth and stress adaptation. However, the molecular mechanisms that promote the early onset of endoreplication in response to DNA damage are still not fully understood.

The onset of endoreplication is controlled by a decrease in mitotic CDK activity, driven by selective degradation of mitotic cyclins. This process is mediated by the E3 ubiquitin ligase anaphase-promoting complex/cyclosome (APC/C) complex (Heyman and De Veylder 2012). The activity of APC/C is determined by its association with activating subunits known as CELL CYCLE SWITCH 52 A (CCS52A) proteins that are functional homologs of mammalian Cdh1 and *Drosophila* Fzr (Tarayre et al. 2004). Among the two CCS52A proteins in Arabidopsis, CCS52A1 is particularly critical for regulating endoreplication; loss of *CCS52A1* reduces DNA ploidy levels, while its overexpression promotes endoreplication (Larson-Rabin et al. 2009). In Arabidopsis roots, *CCS52A1* specifically accumulates in the TZ, where cells initiate longitudinal elongation. This spatial transcriptional regulation of *CCS52A1* is tightly linked to the transition from mitotic cycles to endoreplication, highlighting its importance in root growth (Takahashi et al. 2013; Vanstraelen et al. 2009). Previously, cytokinins have been shown to promote the onset of endoreplication. ARABIDOPSIS RESPONSE REGULATOR 2 (ARR2), an Arabidopsis type-B response regulator activated by cytokinin signaling, directly induces *CCS52A1* expression (Takahashi et al. 2013). This regulatory mechanism facilitates the shift from cell division to endoreplication, emphasizing the role of cytokinin in modulating root development through endoreplication.

In this study, we examined the cellular responses of Arabidopsis roots to gamma radiation. Exposure to gamma rays triggers a range of DDRs including the suppression of cell division, vascular stem cell-specific cell death, and early onset of endoreplication. These responses are primarily mediated by SOG1. Our findings reveal that SOG1 directly regulates the expression of *CCS52A1* by binding to its gene body in response to DSBs. Loss of *SOG1* completely abolishes the early onset of endoreplication, whereas the *ccs52a1* mutant displays partially suppressed DDR phenotypes, including reduced meristem size and delayed transition to endoreplication. These results highlight an important role for CCS52A1 in mediating SOG1-dependent responses to DNA damage. This study provides new insights into the molecular mechanisms of endoreplication underlying root meristem regulation and the adaptive strategies employed by plants to maintain genome integrity under genotoxic stress.

## Materials and methods

### Plant materials and growth conditions

*Arabidopsis thaliana* (accession Col-0) was used in this study. *sog1-101* (Ogita et al. 2018), *ProSOG1:SOG1-Myc* (Yoshiyama et al. 2013), and *ccs52a1-1* (Larson-Rabin et al. 2009) were previously described. *ccs52a1-1 sog1-101* was generated by crossing. Arabidopsis seeds were sown on Murashige and Skoog (MS) plates [1/2 × MS salts, 1% sucrose, 0.5 g L^− 1^ 2-(N-morpholino)ethanesulfonic acid, and 1.2% phyto agar (pH 6.3)]. After incubation at 4 °C for 2 days, the plates were placed vertically under continuous light at 23 °C.

## DNA damage treatment

For gamma ray treatment, five-day-old seedlings were exposed to 50 Gy/h of ^60^Co gamma radiation for 2 h at the Cobalt-60 irradiation facility, Takasaki Institute for Advanced Quantum Science, and were subsequently grown under continuous light conditions. For zeocin or hydroxyurea treatment, seedlings were transferred to MS medium supplemented with 8 µM zeocin (Thermo Fisher Scientific) or 2 mM hydroxyurea (Sigma), respectively, and grown under the same conditions.

## Propidium iodide (PI) staining and measurement of cell death

Roots were stained with 10 µM PI solution for 1 min at room temperature. Root tips were observed using a confocal laser scanning microscope (LSM880; Zeiss). The area of cell death was measured using Fiji image analysis software (http://fiji.sc) by defining the field, in which PI infiltrated cells.

## Measurement of the distance from the QC to the first endoreplicated cells

Roots were fixed with 4% paraformaldehyde in phosphate-buffered saline (PBS, pH 7.4) for 24 h at 4 °C. The samples were washed twice with PBS and stained with 1 mg/L 4’,6-diamidino-2-phenylindole (DAPI). Root tips were subsequently observed using a confocal laser-scanning microscope (LSM880; Zeiss). The distance from the QC to the first endoreplicated cells was measured using Fiji image analysis software. The distance was defined as the length from the epidermal cell at the position of the QC to the first endoreplicated cell in the hair cell layer.

## Quantitative real-time PCR (qRT–PCR)

Total RNA was extracted from roots using the Plant Total RNA Mini Kit (Favorgen). First-strand cDNA was synthesized from total RNA using the ReverTra Ace qPCR RT Master Mix (Toyobo), according to the manufacturer’s instructions. qRT**–**PCR was performed with the KAPA SYBR Fast qPCR Kit (Kapa Biosystems) using 100 nM primers and first-strand cDNAs. The primer sequences are listed in Table S1. PCR reactions were conducted with the PikoReal Real-Time PCR system (Thermo Fisher Scientific) according to the following conditions: 95 °C for 5 min; 50 cycles at 95 °C for 10 s, 55 °C for 15 s, and 72 °C for 30 s. Gene expression was analyzed using the 2^−∆∆Ct^ method (Livak and Schmittgen 2001) and normalized using *ACTIN2* as a reference gene. Three biological replicates were used.

### Chromatin Immunoprecipitation quantitative PCR (ChIP–qPCR)

*pSOG1:SOG1-MYC* transgenic seeds were germinated in liquid MS medium and cultured under continuous light at 23 °C with gentle shaking at 50 rpm. After two weeks, whole seedlings were treated with or without 20 µM zeocin for 2 h and subsequently fixed with 1% formaldehyde for 15 min. The seedlings were ground, and chromatin DNA was extracted. Chromatin DNA was sheared into fragments of 150 to 500 bp by sonication. After sonication, chromatin bound to the SOG1-Myc fusion protein was immunoprecipitated using an anti-Myc antibody (Millipore) (Gendrel et al. 2005). The specific primers listed in Table S1 were used for qRT–PCR to quantify the genomic DNA bound to the precipitated chromatin. PCR was conducted using the PikoReal Real-Time PCR system (Thermo Fisher Scientific) according to the following conditions: 95 °C for 5 min; 70 cycles at 95 °C for 10 s, 60 °C for 15 s, and 72 °C for 30 s. Relative gene expression was analyzed using the 2^−∆∆Ct^ method (Livak and Schmittgen 2001).

## Results

### SOG1 mediates gamma irradiation-induced suppression of cell division and vascular stem cell-specific cell death in Arabidopsis roots

**Fig. 1:**
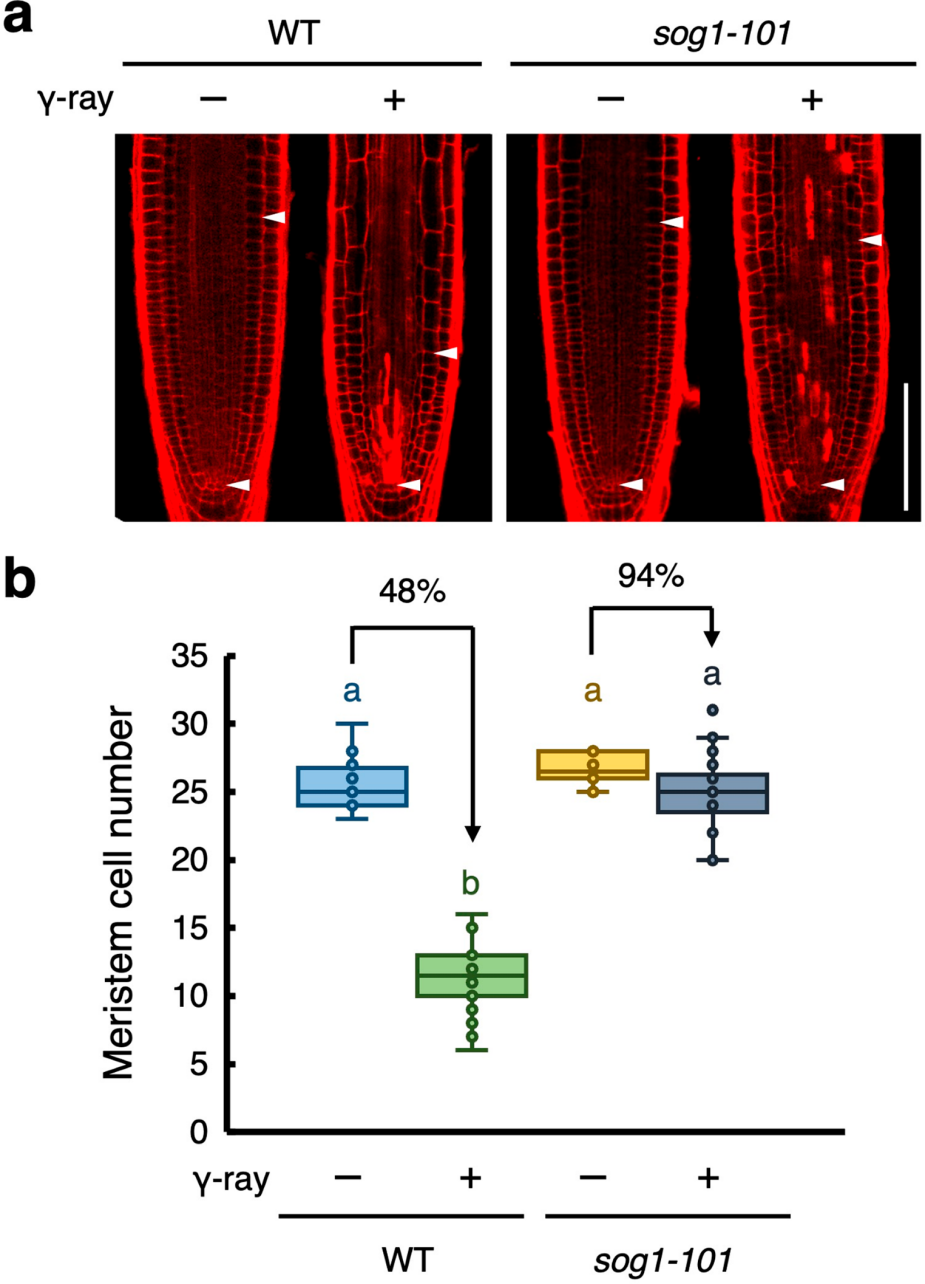
SOG1 is involved in gamma-induced reduction of the meristem size. Five-day-old WT and *sog1-101* seedlings were exposed to gamma radiation at doses of 0 Gy (- γ-ray) or 100 Gy (+ γ-ray), and grown for 24 h. (**a**) Representative images of root meristem. Roots were stained with PI and observed using a confocal laser scanning microscope. Arrowheads indicate the quiescent center (QC) (lower) and boundary between the meristematic and transition zones (upper). Bar = 100 μm. (**b**) The number of cortex cells between the QC and the first elongated cell (*n* > 30). In the box plots, center lines show the medians; box limits indicate the 25th and 75th percentiles; whiskers extend 1.5 times the interquartile range; and circles indicate each value in the data set. Different letters indicate significant difference (*P* < 0.05; Student’s *t*-test)

Ionizing radiation have been widely used as powerful mutagens in plants. Gamma rays are high-energy electromagnetic radiation known to induce DNA damage (e.g., single-strand breaks, DSBs, and base damage) (Kim et al. 2019). To investigate the plant responses to gamma radiation, five-day-old Arabidopsis roots were exposed to 100 Gy of gamma rays, and their root tips were observed at 24 h post-irradiation after staining cell walls with propidium iodide (PI). In wild-type (WT) plants, exposure to gamma rays reduce the size of the root meristem (Fig. 1a). Measurement of cell numbers showed that exposure to gamma rays reduced cell numbers in the cortex cell layer of the root meristem by 48% (Fig. 1b). Cell death was observed in vascular stem cells and their daughters (Figs. 1a, S1a). These findings suggest that exposure to gamma rays suppresses cell division in the root tip.

In response to DNA damage, the transcription factor SOG1 stimulates DNA damage responses in plants (Yoshiyama et al. 2009). To determine whether SOG1 is involved in gamma ray-induced suppression of cell division, we analyzed the responses to gamma radiation in the loss-of-function mutant *sog1-101*. Gamma irradiation did not reduce the size of the root meristem or decreased cortical cell numbers in the *sog1-101* mutant (Fig. 1). This result suggests that SOG1 is involved in suppressing cell division in the root meristem caused by exposure to gamma rays. Furthermore, unlike the WT roots, where cell death specifically occurs in vascular stem cells and their daughters, the *sog1-101* mutant exhibited irregular cell death in root tips (Figs. 1a, S1a). Notably, the area of dying cells in the *sog1-101* mutant was significantly smaller than that in WT plants (Fig. S1b). These findings suggest that SOG1 plays a critical role in mediating the suppression of cell division and induction of vascular stem cell-specific cell death in response to gamma radiation in Arabidopsis roots.

### SOG1 is involved in gamma ray-induced early onset of endoreplication in roots

**Fig. 2:**
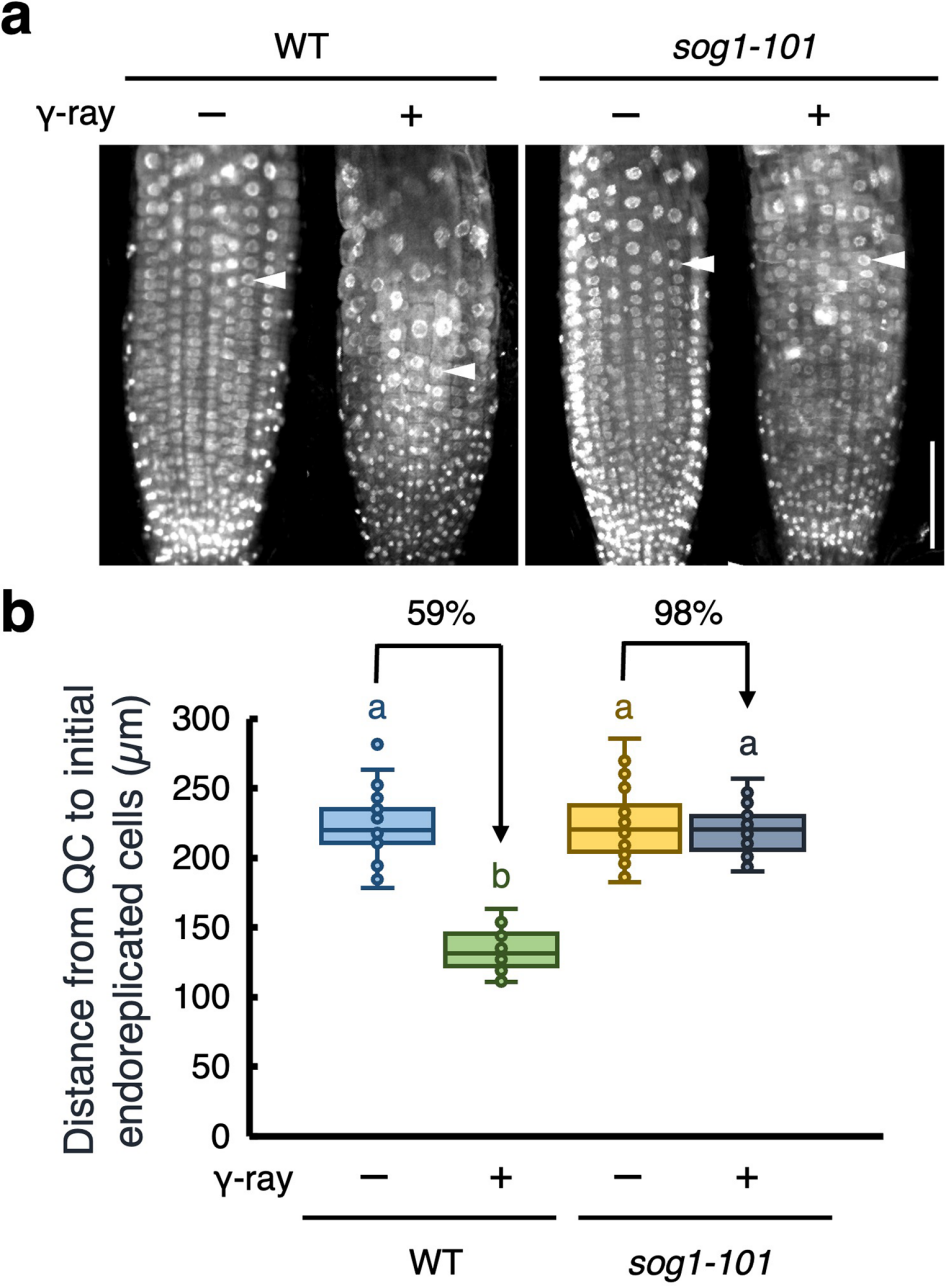
SOG1 regulates early onset of endoreplication under gamma irradiation. (**a**) Representative images of DAPI-stained nuclei of roots. Five-day-old WT and *sog1-101* seedlings were exposed to gamma radiation at doses of 0 Gy (- γ-ray) or 100 Gy (+ γ-ray), and grown for 24 h. Roots were stained with DAPI and observed using a confocal laser scanning microscope. Arrowheads indicate the first endoreplicated nuclei in the epidermal hair cell layer. Bar = 100 μm. (**b**) The distance from the quiescent center (QC) to the first endoreplicated nucleus in individual epidermal hair cell layer (*n* > 22). Different letters indicate significant difference (*P* < 0.05; Student’s *t*-test)

In Arabidopsis roots, DSBs suppress cell division in the root meristem and induce an early transition to endoreplication in the TZ, leading to cell enlargement (Adachi et al. 2011). To investigate whether gamma rays trigger an early onset of endoreplication, we analyzed the nuclear size in root tips exposed to gamma radiation, considering the correlation between ploidy levels and nuclear size (Takahashi et al. 2013). To visualize nuclei, roots were stained with 4’,6-diamidino-2-phenylindole (DAPI), and the nuclear size of cells in the root hair cell files was observed as these cells exhibit distinct timing of endoreplication compared to that by non-root hair cell files. In non-irradiated roots, transition to endoreplication was observed at a distance of 220 μm from the quiescent center (QC) (Fig. 2). In contrast, gamma-irradiated roots exhibited this transition at approximately 130 μm from the QC (Fig. 2). These results indicate that gamma radiation promotes an early onset of endoreplication. Next, to examine whether SOG1 is involved in this gamma-induced early onset of endoreplication, we analyzed *sog1* mutant roots. In the *sog1-101* mutant, gamma radiation did not induce an early transition to endoreplication (Fig. 2). This finding suggests that SOG1 plays a critical role in promoting the early onset of endoreplication in response to gamma radiation in Arabidopsis roots.

### DSBs induce *CCS52A1* expression in a SOG1-dependent manner

**Fig. 3:**
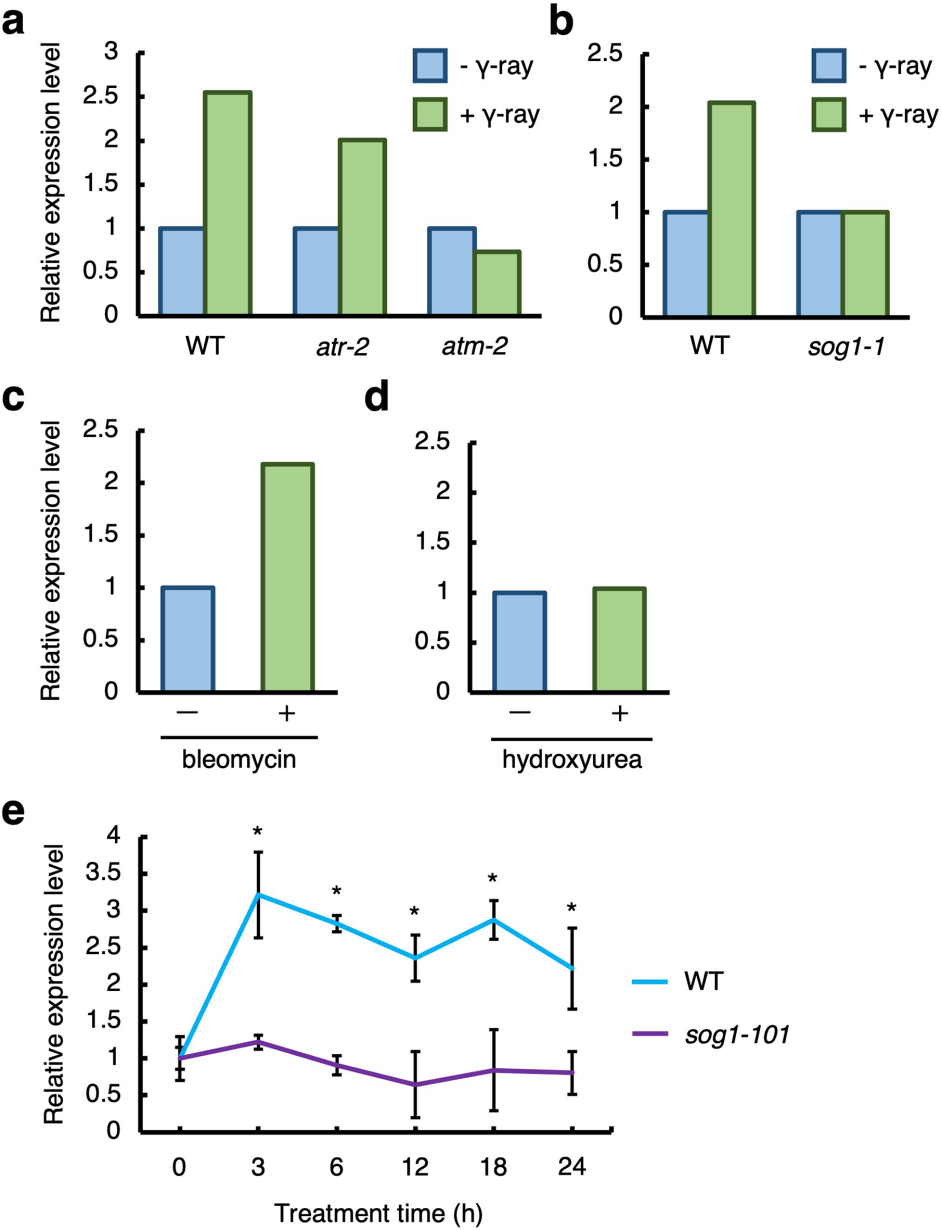
DSBs induce *CCS52A1* in a SOG1-dependent manner. Transcriptional response of *CCS52A1* to gamma irradiation, bleomycin, and hydroxyurea. Data were obtained from the transcriptome results of Culligan et al. (2006) (**a**), Yoshiyama et al. (2009) (**b**), Takahashi et al. (2019) (**c**), and Cools et al. (2010) (**d**). (**a**) Five-day-old WT, *atr-2*, and *atm-2* seedlings were irradiated at 100 Gy gamma ray and harvested at 1.5 h after irradiation for total RNA isolation in two biological repeats. (**b**) Five-day-old WT and *sog1-1* seedlings were gamma-irradiated with 100 Gy and harvested at 1.5 h after irradiation for total RNA isolation in two biological repeats. (**c**) Five-day-old WT seedlings were treated with 0.6 µg/mL bleomycin for 10 h, and total RNA was extracted from root tips in three biological repeats. (**d**) Seven-day-old WT seedlings were treated with 2 mM hydroxyurea for 24 h, and root tips were harvested for RNA extraction in two biological repeats. Relative transcript levels of *CCS52A1* are indicated with that of the control set to 1. (**e**) Transcript levels of *CCS52A1* in the presence of zeocin. Five-day-old WT and *sog1-101* seedlings were transferred onto MS plates supplemented with 8 µM zeocin and grown for 0, 3, 6, 12, 18, and 24 h. Total RNA was extracted from roots and subjected to qRT-PCR. Transcript level of *CCS52A1* was normalized to that of *ACTIN2*, and are indicated as relative values, with the value at 0 h set to 1. Data are presented as mean ± SD calculated from three biological and technical replicates. Significant differences from the 0 h control were determined by Student’s *t*-test: *P < 0.05

CCS52A1, an activator of APC/C, is required for the transition from mitotic cell cycle to endocycle. The expression of *CCS52A1* is shown to be determinant for the onset of endoreplication in Arabidopsis roots (Larson-Rabin et al. 2009; Takahashi et al. 2013). To investigate whether CCS52A1 contributes to the early transition to endoreplication following exposure to gamma radiation, we analyzed *CCS52A1* expression using the transcriptome data from previous studies (Cools et al. 2010; Culligan et al. 2006; Takahashi et al. 2019; Yoshiyama et al. 2009). The results showed that *CCS52A1* expression was upregulated approximately 2.5 and 2-fold after exposure to 100 Gy gamma radiation (Fig. 3a, b). Since ATM, ATR and SOG1 are key components that transmit DNA damage signals downstream, *CCS52A1* expression in *atm-2*, *atr-2* and *sog1-1* mutants was evaluated upon exposure to gamma radiation. Induction of *CCS52A1* expression occurred in the *atr-2* mutant but not in the *atm-2* and the *sog1-1* mutant (Fig. 3a, b), suggesting that ATM and SOG1 is required for gamma radiation-induced *CCS52A1* expression. To further explore whether *CCS52A1* induction occurs under other types of DNA damage, the effects of bleomycin that induces DSBs (Berdy 1980), and hydroxyurea (HU) that inhibits deoxyribonucleotide production and induces replication stress (Saban and Bujak 2009; Wang and Liu 2006), were analyzed. The result showed that *CCS52A1* expression was induced by bleomycin treatment but not with HU treatment (Fig. 3c, d). This indicates that *CCS52A1* transcription is induced via the ATM-SOG1 pathway in response to DSBs. Furthermore, consistent with the lack of *CCS52A1* induction, promotion of early onset of endoreplication was not observed in root tips after HU treatment (Fig. S2).

To investigate the temporal dynamics of *CCS52A1* expression in response to DSBs, seedlings were treated with zeocin, a DSB-inducing agent (Berdy 1980). Five-day-old WT seedlings were transferred to medium containing 8 µM zeocin, and total RNA was extracted from root tips after 3, 6, 12, 18, and 24 h treatment. Quantitative RT-PCR (qRT-PCR) analysis revealed that *CCS52A1* expression increased approximately threefold after 3 h of zeocin treatment, compared to that in untreated controls, and remained elevated thereafter (Fig. 3d). These results suggest that *CCS52A1* rapidly responds to DSBs. In contrast, no induction of *CCS52A1* expression was observed in the *sog1-101* mutant after zeocin treatment (Fig. 3d), indicating that the ATM-SOG1 pathway plays a crucial role in the early induction of *CCS52A1* transcription in response to DSBs.

### SOG1 binds to the *CCS52A1* locus in response to DSBs

**Fig. 4:**
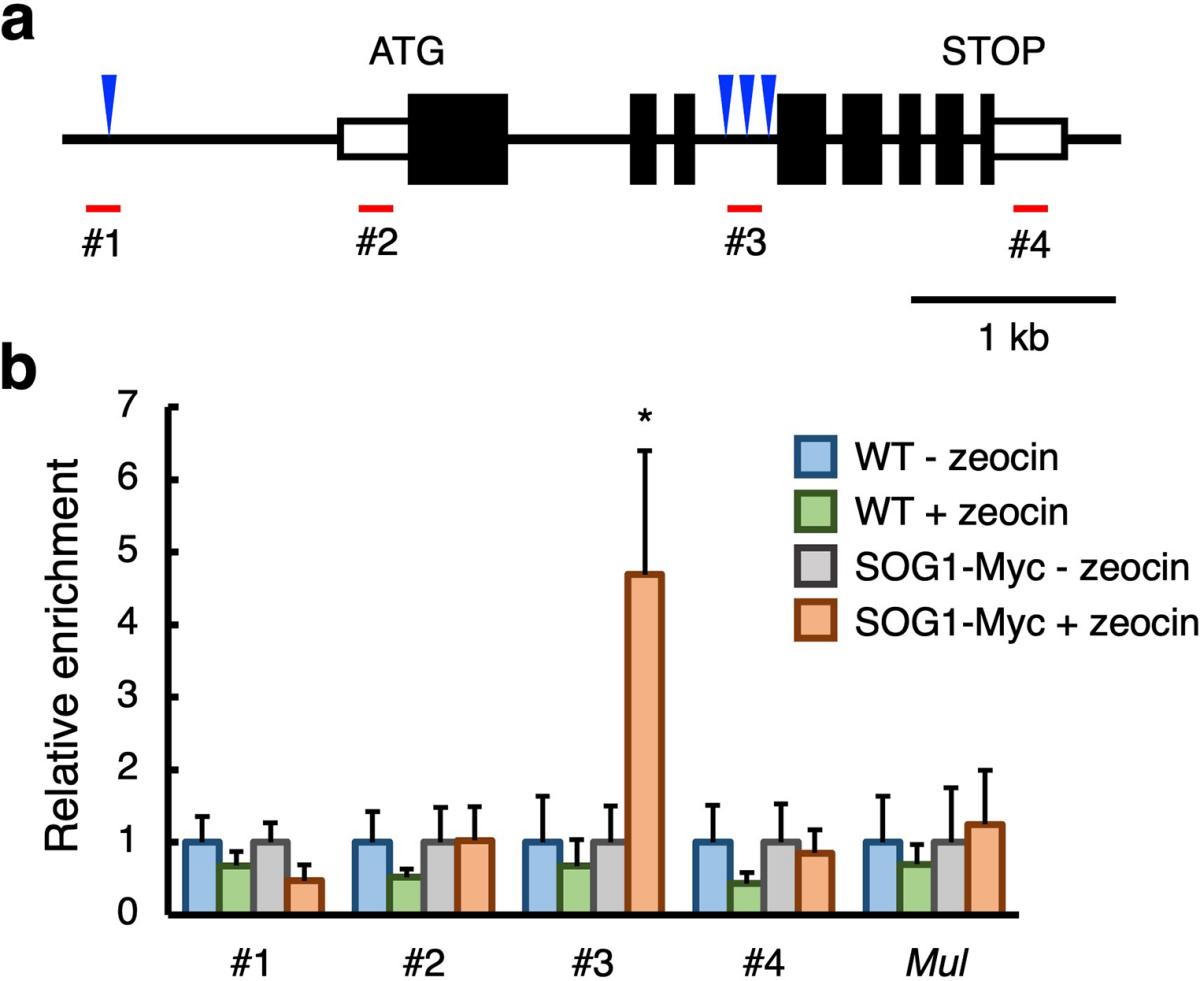
SOG1 directly binds to the *CCS52A1* locus. (**a**) Schematic representation of the *CCS52A1* genomic region. Black and white boxes indicate the coding region and untranslated regions (UTRs), respectively. Blue triangles and red lines represent the locations of the SOG1-binding motifs and regions amplified by chromatin immunoprecipitation quantitative PCR **(**ChIP–qPCR), respectively. (**b**) Identification of SOG1-binding sites. Two-week-old WT and *pSOG1:SOG1-Myc* (SOG1-Myc) seedlings grown in liquid MS medium were further cultured in a medium with (+ zeocin) or without (− zeocin) 20 µM zeocin for 2 h. Genomic DNA was extracted from whole seedlings, and chromatin bound to SOG1-Myc was immunoprecipitated. qPCR was conducted to amplify the *CCS52A1* genomic regions shown in (**a**); the promoter (#1), 5ʹ-UTR (#2), the third intron (#3), and 3ʹ-UTR (#4). *Mutator-like transposon* (*Mul*) was used as a negative control. Fold enrichments of DNA fragments by ChIP–qPCR are indicated as relative values, with that for the WT (- zeocin) set to 1. Data are presented as mean ± SD (*n* = 3) calculated from three biological replicates. For the data of SOG1-Myc, significant differences from the WT were determined by Student’s *t*-test: *P < 0.05

The above findings prompted us to investigate whether SOG1 directly regulates *CCS52A1* expression under DSBs. A search for the SOG1-binding consensus motif, CTT(N)_7_AAG, within the *CCS52A1* genomic region revealed one motif (*CTT*CTCCAGT*AAG*) in the promoter region approximately 1-kb upstream from the start codon, and three consensus motifs (*CTT*TATCGAG*AAG*, *CTT*TGTGGTT*AAG*, and *CTT*GGCGGAT*AAG*) within the third intron, respectively (Fig. 4a; regions #1, #3). To determine whether SOG1 binds to the *CCS52A1* locus, chromatin immunoprecipitation (ChIP) assays were performed using transgenic plants harboring *pSOG1:SOG1-Myc*. DNA fragments bound by SOG1 were analyzed by qPCR using primers targeting genomic regions in the promoter, 5′-untranslated region (UTR), third intron, and 3′-UTR (Fig. 4a, regions #1–4). Under normal growth condition, no significant SOG1 binding was detected in any of these regions (Fig. 4b). However, after seedlings were treated with 20 µM zeocin for 2 h to induce DSBs, SOG1-bound DNA fragments were significantly enriched in the third intron (region #3) that contains three SOG1-binding motifs (Fig. 4b). These findings suggest that SOG1 binds to the *CCS52A1* locus in response to DSBs, facilitating the transcriptional induction of *CCS52A1*.

### CCS52A1 is partially involved in gamma ray-induced early onset of endoreplication in roots

**Fig. 5:**
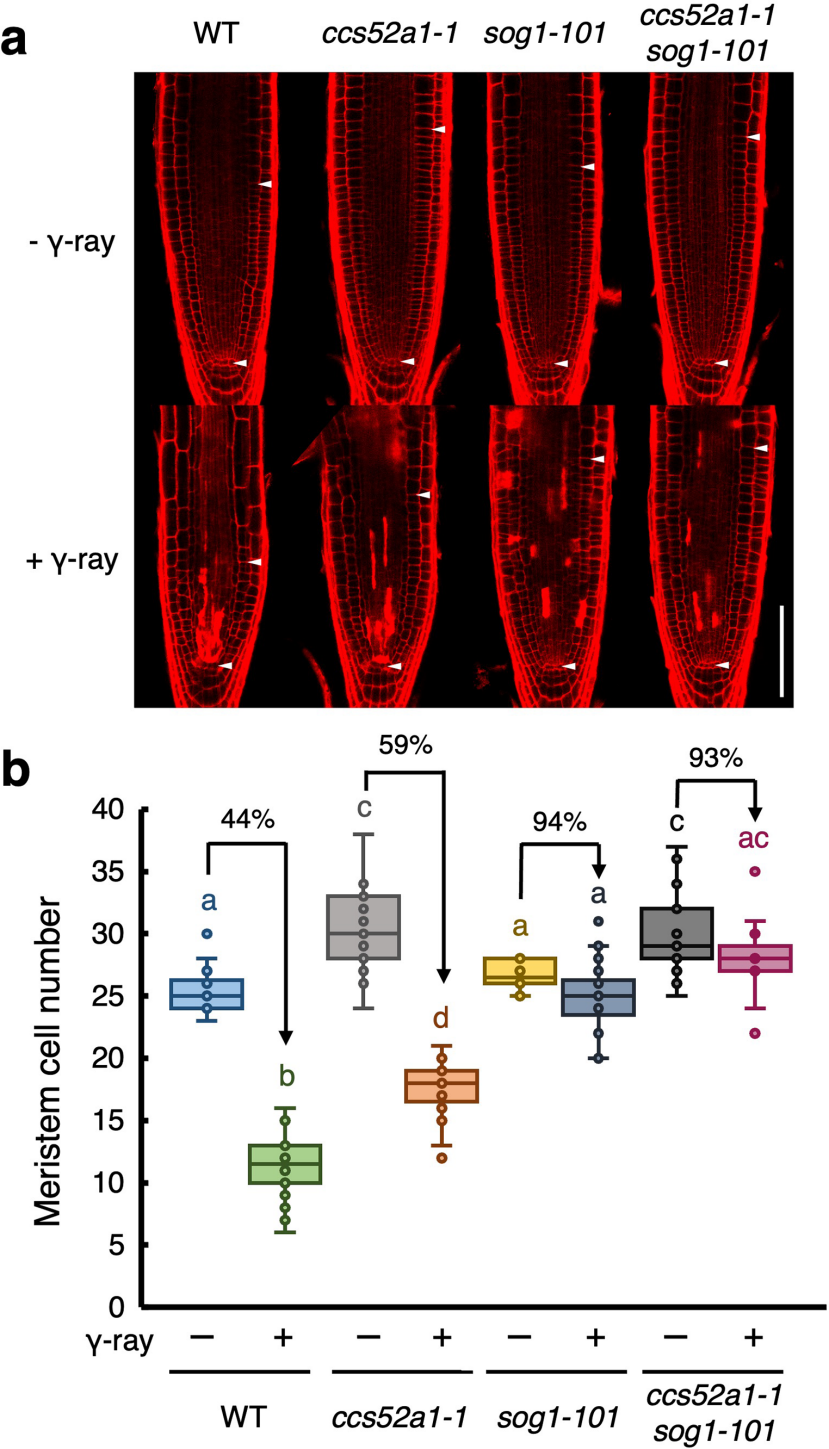
CCS52A1 is partially required for gamma-induced reduction of the meristem size. Five-day-old WT, *ccs52a1-1*, *sog1-101*, and *ccs52a1-1 sog1-101* seedlings were exposed to gamma radiation at doses of 0 Gy (- γ-ray) or 100 Gy (+ γ-ray) and grown for 24 h. (**a**) Representative images of root meristem. Roots were stained with PI and observed using a confocal laser scanning microscope. Arrowheads indicate the quiescent center (QC) (lower) and boundary between the meristematic and transition zones (upper). Bar = 100 μm. (**b**) The number of cortex cells between the QC and the first elongated cell (*n* > 17). In the box plots, center lines show the medians; box limits indicate the 25th and 75th percentiles; whiskers extend 1.5 times the interquartile range; and circles indicate each value in the data set. Different letters indicate significant difference (*P* < 0.05; Student’s *t*-test)

**Fig. 6:**
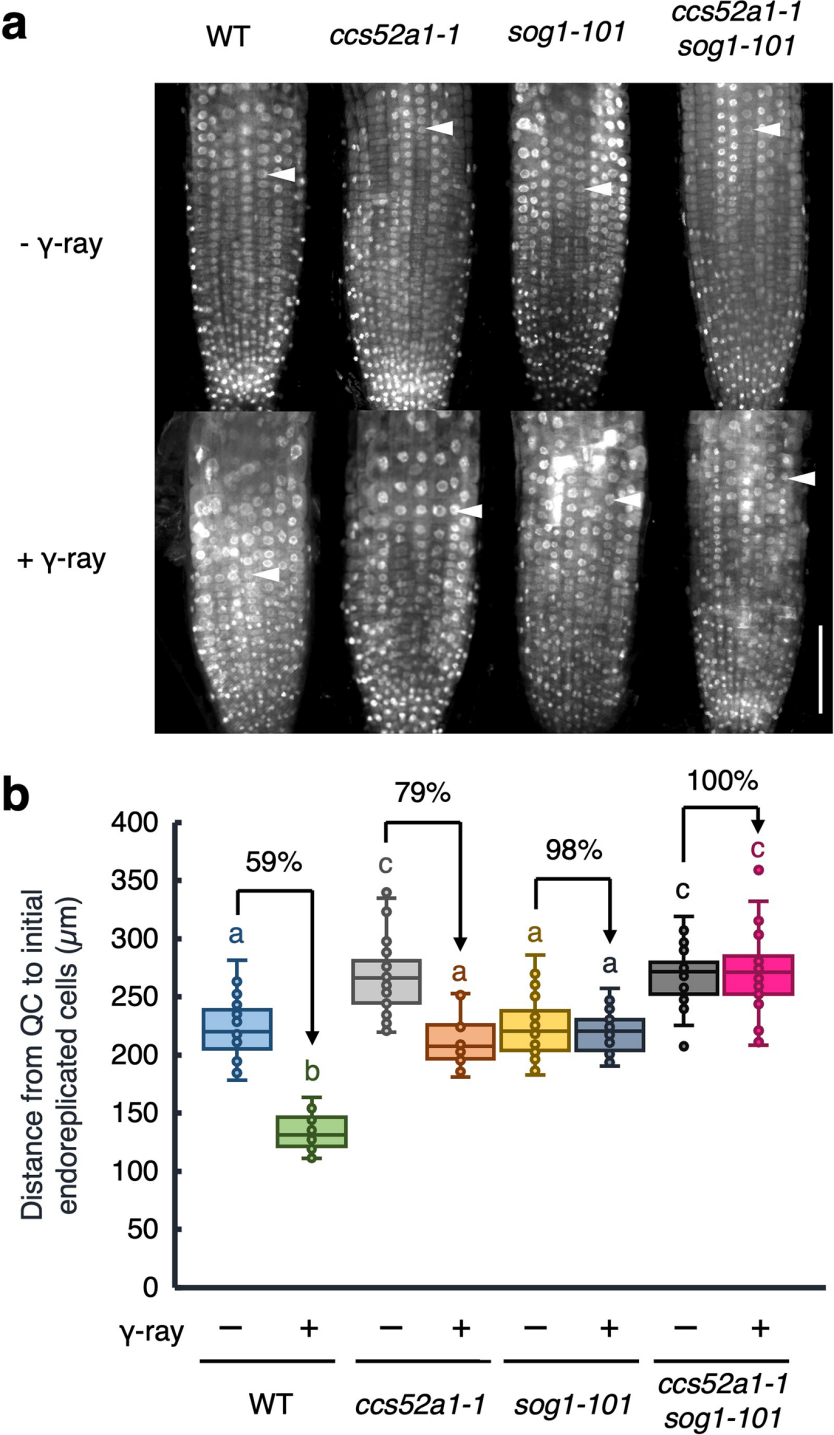
The SOG1–CCS52A1 pathway partially participates in early onset of endoreplication under gamma irradiation. (**a**) Representative images of DAPI-stained nuclei of roots. Five-day-old WT, *ccs52a1-1*, *sog1-101*, and *ccs52a1-1 sog1-101* seedlings were exposed to gamma radiation at doses of 0 Gy (- γ-ray) or 100 Gy (+ γ-ray), and grown for 24 h. Roots were stained with DAPI and observed using a confocal laser scanning microscope. Arrowheads indicate the first endoreplicated nuclei in the epidermal hair cell layer. Bar = 100 μm. (**b**) The distance from the quiescent center (QC) to the first endoreplicated nucleus in individual epidermal hair cell layer (*n* > 20). Different letters indicate significant difference (*P* < 0.05; Student’s *t*-test)

*CCS52A1* expression is induced in a SOG1-dependent manner upon DSBs (Fig. 3d), suggesting the role of CCS52A1 in the early onset of endoreplication in roots in response to DSBs. To investigate this, we first analyzed the response of the *ccs52a1* knockout mutant to gamma radiation by observing the phenotype of root tips after exposure to 100 Gy of gamma rays. The untreated *ccs52a1-1* mutant exhibited an expanded meristematic zone compared to the WT (Fig. 5a), with an increased number of cortex cells in the root meristem (Fig. 5b), consistent with previous reports (Takahashi et al. 2013). After exposure of gamma rays, the number of cortex cells in the root meristem of WT plants decreased by 44%, whereas this reduction was attenuated to 59% in the *ccs52a1-1* mutant (Fig. 5b). Note that no significant difference in the cell death area was observed between the WT and *ccs52a1-1* mutant plants (Fig. S1), suggesting that cell death does not account for the meristem size in the *ccs52a1-1* mutant. Next, to assess whether the relatively low reduction of meristem size in the *ccs52a1-1* mutant was associated with altered onset of endoreplication, we measured the distance between the QC and the first endoreplicated cells. In WT plants, gamma radiation reduced the distance to endoreplicated cells by 59% compared to that in non-irradiated roots. However, in the *ccs52a1-1* mutant, the reduction was attenuated by 79% (Fig. 6). This result indicates that CCS52A1 contributes to the early onset of endoreplication induced by DSBs.

To explore the genetic relationship between *CCS52A1* and *SOG1*, we generated a *ccs52a1-1 sog1-101* double mutant and analyzed its response to gamma radiation. In the *sog1-101* mutant, no early onset of endoreplication occurred after exposure to gamma radiation (Fig. 6). Similarly, the *ccs52a1-1 sog1-101* double mutant showed no significant changes in root meristem size and cortex cell number after exposure to gamma irradiation (Fig. 5). Furthermore, no early onset of endoreplication was observed in the double mutant upon exposure to gamma rays (Fig. 6). These results suggest that SOG1 acts upstream of CCS52A1 and plays a predominant role in regulating the early onset of endoreplication triggered by gamma ray-induced DSBs in roots.

### CDK activity is reduced in the *ccs52a1* mutant after DSBs treatment

**Fig. 7:**
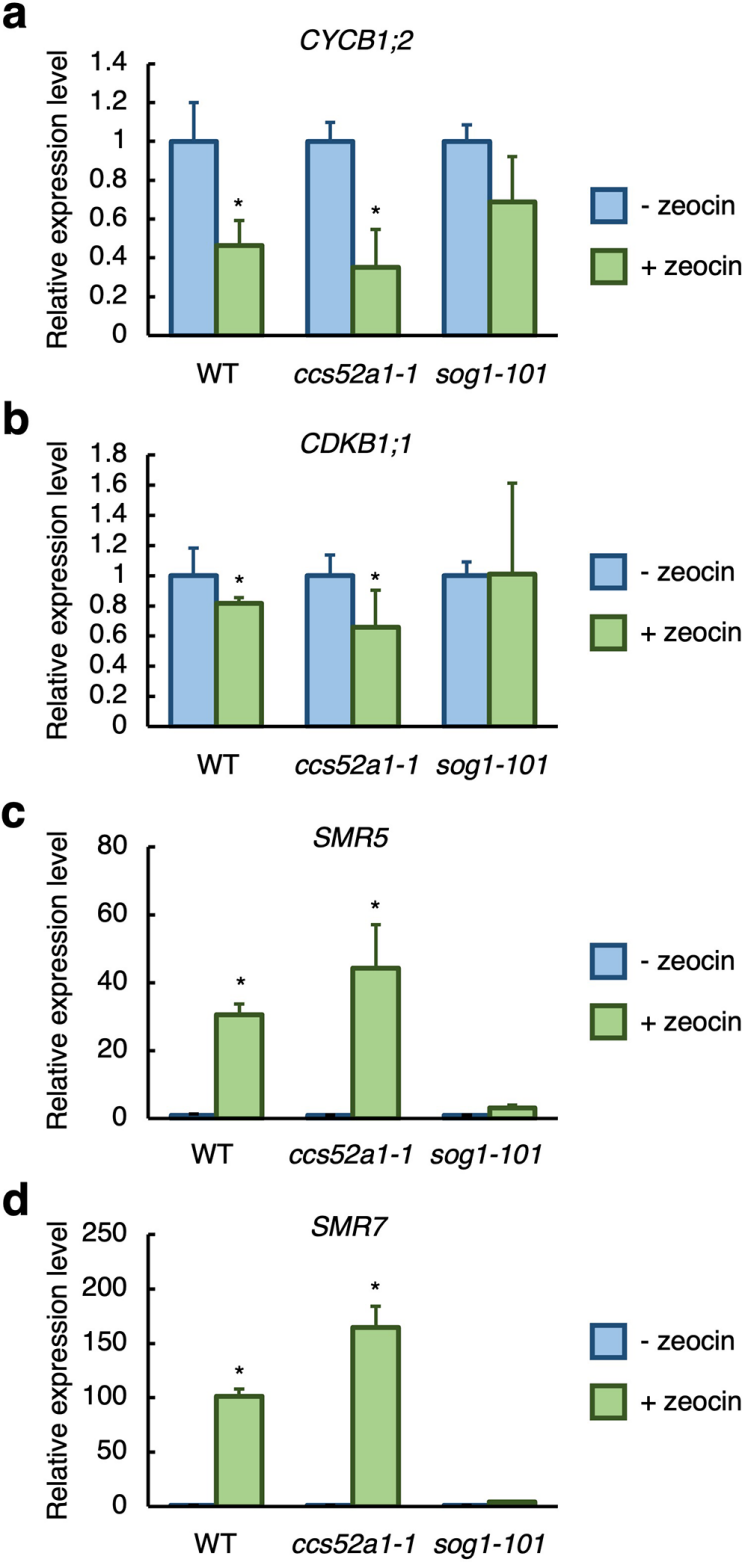
CCS52A1 is not involved in controlling the expression of *CYCB1;2*, *CDKB1;1*, *SMR5*, and *SMR7* under DNA damage. Transcript levels of *CYCB1;2*, *CDKB1;1*,* SMR5*, and *SMR7*. Five-day-old WT, *ccs52a1-1* and *sog1-101* seedlings were transferred onto MS plates supplemented with (+ zeocin) or without (- zeocin) 8 µM zeocin and grown for 24 h. Total RNA was extracted from root tips and subjected to qRT-PCR. Transcript levels of *CYCB1;2*, *CDKB1;1*,* SMR5*, and *SMR7* were normalized to that of *ACTIN2*, and are indicated as relative values, with that for the control (- zeocin) set to 1. Data are presented as mean ± SD calculated from three biological and technical replicates. Significant differences from the control were determined by Student’s *t*-test: *P < 0.05

As mentioned above, unlike the *sog1* mutant that exhibited no early onset of endoreplication after gamma radiation, the early transition to endoreplication partially occurred in the *ccs52a1* mutant (Fig. 6). This observation suggests a possibility that other factor(s) participate in the early onset of endoreplication upon DSBs. The onset of endoreplication is controlled by a reduction in mitotic CDK activity (De Veylder et al. 2011). This implies that in the *ccs52a1* mutant, a decrease in CDK activity triggered by DSBs may lead to a relatively early transition to endoreplication. Previous studies have reported that exposure to DSB-inducing treatments leads to decreased expression of *CDKs* and several cyclin genes, and induction of CDK inhibitor genes (Adachi et al. 2011; Culligan et al. 2006; Yi et al. 2014). Therefore, we investigated the expression of these genes in the *ccs52a1* and *sog1* mutants after DSB treatment. Five-day-old seedlings of the *ccs52a1-1* and *sog1-101* mutants were treated with 8 µM zeocin for 24 h, and total RNA was extracted from the root tips. The expression of *CYCB1;2* and *CDKB1;1*, which are known to be downregulated upon DSB-inducing treatment, and that of the CDK inhibitors *SMR5* and *SMR7*, which are upregulated in response to DSBs (Adachi et al. 2011; Culligan et al. 2006; Yi et al. 2014), were analyzed. In the WT, consistent with previous reports, zeocin treatment resulted in decreased expression of *CYCB1;2* and *CDKB1;1*, and increased expression of *SMR5* and *SMR7* (Fig. 7), while no significant changes in the expression of any of these genes were observed in the *sog1-101* mutant (Fig. 7), suggesting that the reduction in CDK activity induced by DSBs occur in a SOG1-dependent manner. In contrast, the *ccs52a1-1* mutant exhibited a gene expression profile similar to that of the WT, with reduced expression of *CYCB1;2* and *CDKB1;1*, and increased expression of *SMR5* and *SMR7* after zeocin treatment (Fig. 7). These results indicate that DSB-induced reduction of CDK activity occurs in the *ccs52a1* mutant but not in the *sog1* mutant. Therefore, the reduction in CDK activity in the *ccs52a1* mutant upon DSBs treatment may underlie the phenotypic differences between the *ccs52a1* and *sog1* mutants, particularly the partial induction of an early transition to endoreplication observed in the *ccs52a1* mutant, whereas the *sog1* mutant exhibits complete suppression of this transition.

## Discussion

DNA damage profoundly affects cellular survival and genomic stability, making the maintenance of genome integrity essential for all living organisms. The DNA damage response pathway plays a crucial role in preserving genomic integrity by repairing DNA lesions that, if left unrepaired, can lead to genome instability. This repair process is often accompanied by a transient arrest of the cell cycle, which allows sufficient time for the repair of DNA damage before cells enter into mitosis. In plants, the NAC-type transcription factor SOG1 serves as a central regulator of the DDR, orchestrating the expression of numerous target genes involved in DNA repair, cell death, and cell cycle regulation (Yoshiyama et al. 2009). In this study, we revealed that SOG1 directly induces the expression of *CCS52A1*, an activator of the E3 ubiquitin ligase APC/C, promoting the transition to endoreplication from the mitotic cell cycle in response to DSBs. The APC/C^CCS52A1^ complex ubiquitinates mitotic cyclins, such as CYCB1;1, CYCB1;2, CYCA2;3, and CYCA3;1, leading to their degradation via the 26 S proteasome (Boudolf et al. 2009; Kasili et al. 2010; Mathieu-Rivet et al. 2010). Promotion of mitotic cyclin degradation suppresses CDK activity, thereby facilitating the early onset of endoreplication in response to DSBs.

In this study, we demonstrated that CCS52A1 plays a key role in the early onset of endoreplication in response to gamma irradiation in Arabidopsis roots. Notably, while the *sog1* mutant failed to undergo early onset of endoreplication after gamma radiation, the *ccs52a1* mutant exhibited partial induction of this process. This observation indicates that although CCS52A1 is important for triggering early onset of endoreplication in response to DSBs, additional factors must also contribute to this process. We revealed that in the *ccs52a1* mutant, the expression levels of cell cycle regulators, such as *CDKB1;1*, *CYCB1;2*, *SMR5*, and *SMR7*, were altered in response to DSBs at levels comparable to those observed in the WT. These results suggest that a reduction in CDK activity may partially contribute to the early onset of endoreplication observed in the *ccs52a1* mutant. In addition, it has been shown that Arabidopsis possesses two CCS52A isoforms, CCS52A1 and CCS52A2 (Vanstraelen et al. 2009). Our results showed that *CCS52A2* expression was not induced by zeocin treatment in the WT (Fig. S3a) and remained unchanged in the *ccs52a1* mutant under the same conditions (Fig. S3b). These findings suggest that *CCS52A2* expression is not transcriptionally regulated in response to DNA damage, making it unlikely that CCS52A2 plays a redundant role with CCS52A1 in the SOG1-mediated DNA damage response. However, given that CCS52A2 is a functional homolog of CCS52A1 (Vanstraelen et al. 2009), it remains possible that CCS52A2 may partially compensate for the loss of CCS52A1 function. Further studies, such as analyzing the phenotype of the *ccs52a1 ccs52a2* double mutant in response to DSBs, would be necessary to clarify the extent of functional redundancy between CCS52A1 and CCS52A2.

We revealed that SOG1 binds to the third intron of the *CCS52A1* locus. The mechanism by which SOG1 binding to the intron region of *CCS52A1* activates its gene expression is still elusive. However, it is possible that SOG1 binding facilitates chromatin remodeling, enabling the recruitment of transcriptional machinery necessary for gene activation. Intron regions are increasingly recognized as regulatory hotspots that can influence gene expression through the formation of enhancer-like elements or by modulating chromatin accessibility. Additionally, intron-mediated enhancement (IME) is a known phenomenon in plants, where intronic sequences enhance transcriptional activity (Parra et al. 2011; Rose et al. 2016). SOG1 binding to the *CCS52A1* intron may serve a similar role, promoting efficient transcription in response to DNA damage. Further studies, including chromatin accessibility and the identification of co-factors recruited by SOG1 at the intronic region, will be necessary to elucidate the precise mechanism underlying this regulation.

The extent of endoreplication is regulated by environmental factors. In Arabidopsis, hypocotyl cells rapidly elongate by inducing endoreplication under dark conditions (Gendreau et al. 1999; Kudo and Mii 2004), and nitric acid promotes endoreplication in cotyledons and leaves (Moreno et al. 2020). Additionally, endoreplication is promoted at the pathogen infection sites and during nodule formation in symbiosis (Chandran et al. 2010; Vinardell et al. 2003), and herbivory leads to endoreplication in Arabidopsis (Mesa et al. 2019). These observations suggest that environmental factors usually increase the level of DNA ploidy by stimulating endoreplication. Previously, it has been reported that increased ploidy levels confer distinct advantages under various stress conditions. Under water-deficient conditions, both cell size and DNA content are reduced, causing a decrease in the final leaf size in WT Arabidopsis plants. In contrast, transgenic plants with increased ploidy levels show reduced sensitivity to water deficiency, maintaining leaf growth rates and cell sizes even under stress conditions (Cookson et al. 2006). Similarly, acute UV-B exposure leads to reduced cell proliferation and cell size in Arabidopsis. Mutant deficient in E2Fe/DEL1, a regulator of endoreplication, exhibits less reduction in leaf size than do the control plants. This phenotype is correlated with ploidy level, suggesting that *e2fe/del1* mutant plants utilize the growth capacity of their polyploid cells to compensate for the reduced cell number (Radziejwoski et al. 2011). In this study, we demonstrated that DSBs induced by gamma radiation trigger an early transition to endoreplication in root tips through the SOG1-CCS52A1 pathway. This transition leads to proportional increases in DNA content and cell volume. These findings suggest that under stress conditions limiting cell proliferation, plants might employ a polyploidy-dependent growth compensation mechanism by inducing endoreplication to increase cell volume.

A previous study has shown that cytokinin regulates the onset of endoreplication in Arabidopsis roots. The cytokinin-activated transcription factor ARR2 binds to and activates the *CCS52A1* promoter, thereby inducing the onset of endocycle in Arabidopsis roots (Takahashi et al. 2013). Interestingly, in response to DSBs, the expression of several cytokinin biosynthesis genes, such as *LOG7* and *CYP735A2*, is induced 12 h after zeocin treatment, leading to elevated endogenous cytokinin levels and the activation of cytokinin signaling in Arabidopsis roots (Takahashi et al. 2021). In this study, we found that *CCS52A1* expression was rapidly induced as early as 3 h after DSB treatment in a SOG1-dependent manner. These findings suggests that the early transition to endoreplication in response to DSBs is regulated by the SOG1–CCS52A1 pathway, while subsequent increases in ploidy levels are likely mediated by cytokinin signaling. In addition, zeocin treatment of the *log7-1* mutant showed that *CCS52A1* expression remained elevated even after 24 and 48 h (Fig. S4), suggesting that the direct transcriptional induction of *CCS52A1* by SOG1 may persist beyond the early phase of the DSBs response. These two regulatory modes likely play complementary roles as part of the plant’s survival strategy in response to genotoxic stress. The direct regulation by SOG1 may provide a rapid and efficient mechanism to trigger endoreplication immediately after DNA damage, enabling the plant to quickly adapt to acute stress. On the other hand, the cytokinin-mediated indirect regulation may sustain or amplify this response over time, preparing the plant for prolonged or recurring damage. This dual regulatory system may enhance the plant’s resilience and adaptability under fluctuating environmental conditions.

Auxin is shown to negatively regulate the onset of endoreplication. Mutants with defective auxin signaling, biosynthesis, or transport exhibit an accelerated transition from the mitotic cell cycle to endoreplication, resulting in increased ploidy levels (Ishida et al. 2010). In addition, a previous study has demonstrated that auxin levels decline in response to DSBs in Arabidopsis root tips (Takahashi et al. 2021). These findings suggest that auxin may regulate endoreplication by modulating factors involved in its control. Ethylene, another plant hormone, is also probably required for controlling endoreplication in response to environmental stimuli. In cotyledons of cucumber (*Cucumis sativus*), a UV-B-induced increase in DNA ploidy levels in trichome socket cells is mediated by ethylene biosynthesis (Yamasaki et al. 2010). Additionally, treatment of Arabidopsis hypocotyls with 1-aminocyclopropane-1-carboxylic acid, a precursor of ethylene, induces additional endocycles, while mutants with reduced sensitivity to ethylene display slightly decreased ploidy levels (Gendreau et al. 1999). These findings indicate that ethylene positively affects the regulation of endoreplication; however, the underlying mechanisms remain elusive. Biosynthesis, transport, and signaling of plant hormones exhibit dynamic changes at the tissue and cellular levels in response to environmental conditions. Understanding the mechanisms, by which plant hormones regulate endoreplication, is anticipated to shed light on the role of endoreplication in facilitating plant growth and survival strategies under fluctuating environmental conditions.

## Electronic supplementary material

Below is the link to the electronic supplementary material.

Supplementary Material 1

## Data Availability

The AGI numbers of *SOG1*, *CCS52A1*, *CYCB1;2*, *CDKB1;1*, *SMR5*, and *SMR7* are AT1G25580, AT4G22910, AT5G06150, AT3G54180, AT1G07500, and AT3G27630, respectively. The data that support the findings of our study are available from the corresponding author upon request.
