# Correction: SUPPRESOR OF GAMMA RESPONSE 1 promotes early onset of endoreplication upon DNA double-strand breaks by inducing *CCS52A1* expression in Arabidopsis roots

**DOI:** 10.1007/s10265-025-01647-3

**Published:** 2025-06-03

**Authors:** Toshiki Wada, Ayako N. Sakamoto, Masaaki Umeda, Naoki Takahashi

**Affiliations:** 1 Department of Life Sciences, School of Agriculture, Meiji University, Kawasaki, Kanagawa 214-8571 Japan; 2 Department of Quantum-Applied Biosciences, National Institutes for Quantum Science and Technology, Takasaki, Gumma 370-1292 Japan; 3 Graduate School of Science and Technology, Nara Institute of Science and Technology, Takayama 8916-5, Ikoma, Nara 630-0192 Japan

**Correction: Journal of Plant Research**

10.1007/s10265-025-01630-y

In this article, the author “Masaaki Umeda” was missing from the author group.

The author, Masaaki Umeda is affiliated to “Graduate School of Science and Technology, Nara Institute of Science and Technology, Takayama 8916-5, Ikoma, Nara 630 − 0192, Japan”

The original article has been corrected.

