# Supplementary material for: SUPPRESOR OF GAMMA RESPONSE 1 promotes early onset of endoreplication upon DNA double-strand breaks by inducing CCS52A1 expression in Arabidopsis roots
Source: J Plant Res. 2025 Apr 7;138(4):679–93. doi: 10.1007/s10265-025-01630-y (PMC12238086; doi:10.1007/s10265-025-01630-y)
Supplement: Supplementary file 1 — Supplementary Material 1 [file 10265_2025_1630_MOESM1_ESM.pdf]

Electronic supplementary materials

Title:

SUPPRESOR OF GAMMA RESPONSE 1 promotes early onset of endoreplication upon DNA double-strand breaks by inducing *CCS52A1* expression in Arabidopsis roots

Authors:

Toshiki Wada[1], Ayako N. Sakamoto[2], Naoki Takahashi[1]

Affiliation:

[1]Department of Life Sciences, School of Agriculture, Meiji University, Tama-ku, Kawasaki, Kanagawa 214-8571, Japan.

[2]Department of Quantum-Applied Biosciences, National Institutes for Quantum Science and Technology Takasaki, Gumma 370-1292, Japan.

Journal:

Journal of Plant Research

Corresponding author

Naoki Takahashi

Department of Life Sciences, School of Agriculture, Meiji University

1-1-1 Higashi-mita, Tama-ku, Kawasaki, Kanagawa 214-8571, Japan

Tel.: +81-44-934-7036; Fax +81-44-934-7036

E-mail: takahashi@meiji.ac.jp

Contents:

Table S1

Fig. S1-4

Table S1. Primers for ChIP–qPCR and qRT–PCR

ChIP–qPCR

CCS52A1 #1	5’-AACAGAATTTTCCACCTACACATC-3’<br>5’-CAAGTTGCAATGAAGGTGGTG-3’
CCS52A1 #2	5’-TTAGGCCCATCTTCAAGCCC-3’<br>5’-AGCGGTGTGATTCAGGTGAG-3’
CCS52A1 #3	5’-TCGTGGACTGTAGGATTCTTGC-3’<br>5’-TTCAGGGCACTTTCCAAATACT-3’
CCS52A1 #4	5’-TGATTTCTTCAGCAGCAGCC-3’<br>5’-AGATCTTGTTGCATCCGTGTG-3’
Mul	5’-GATTTACAAGGAATCTGTTGGTGGT-3’<br>5’-CATAACATAGGTTTAGAGCATCTGC-3’

qRT–PCR

CCS52A1	5’-CACGCTGCAAGAGAACAAGA-3’<br>5’-ACCACTTGAGTCCGCATACC-3’
CYCB1;2	5’-CGTACCTGAACAAGTCAGAG-3’<br>5’-CAAGATTTCCGATGTCACCG-3’
CDKB1;1	5’-AGTCCAGAAACCAACACAG-3’<br>5’-CAACAAGCTTACCAGTTCC-3’
SMR5	5’-AACTACGACGACGGAGATACG-3’<br>5’-GCAACTAGGTTGCCGCTTG-3’
SMR7	5’-GCCAAAACATCGATTCGGGC-3’<br>5’-CTCCGGAGTCTTTGCTCCTC-3’
CCS52A2	5’-CTGTGAACACGCCGCAGCAGTG-3’<br>5’-ATCAGCAGTGCCACCACCAGAAG-3’
ACTIN2	5’-CTGGATCGGTGGTTCCATTC-3’<br>5’-CCTGGACCTGCCTCATCATAC-3’

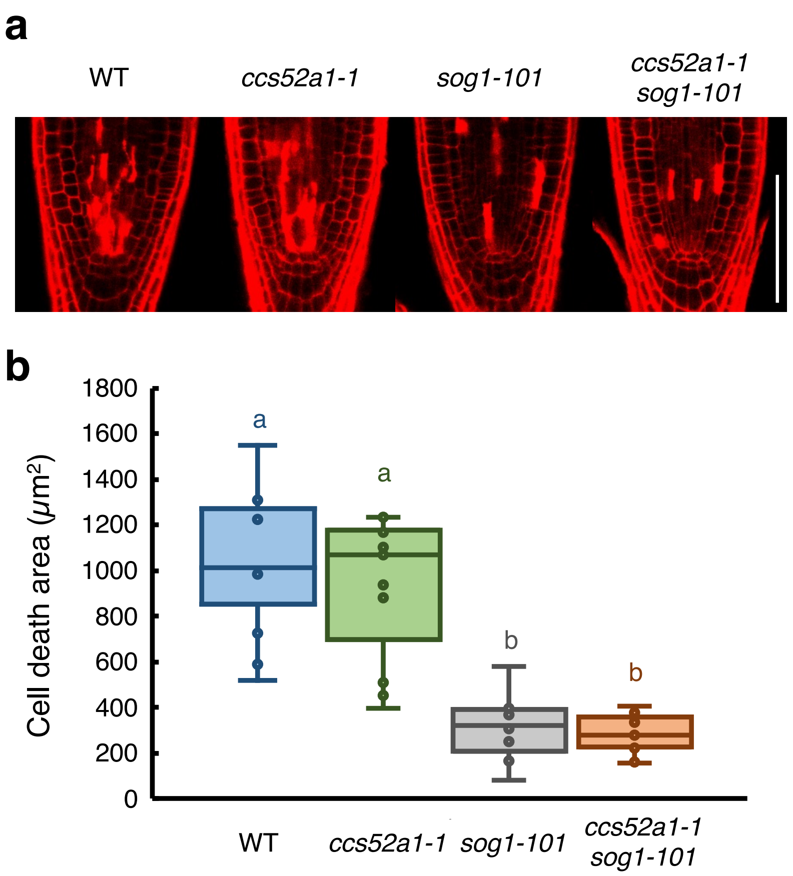

Fig. S1 Cell death area in vascular stem cells and their daughters after gamma irradiation. Five-day-old WT, *ccs52a1-1*, *sog1-101*, and *ccs52a1-1 sog1-101* seedlings were exposed to 100 Gy of gamma rays and grown for 24 h. (**a**) Root tips were observed after staining with propidium iodide (PI). Bar = 100 µm. (**b**) The area of PI-stained dead cells in the root tip (n > 10). In the box plots, center lines show the medians; box limits indicate the 25th and 75th percentiles; whiskers extend 1.5 times the interquartile range; and circles indicate each value in the data set. Different letters indicate significant difference ($P < 0.05$; Student's *t*-test).

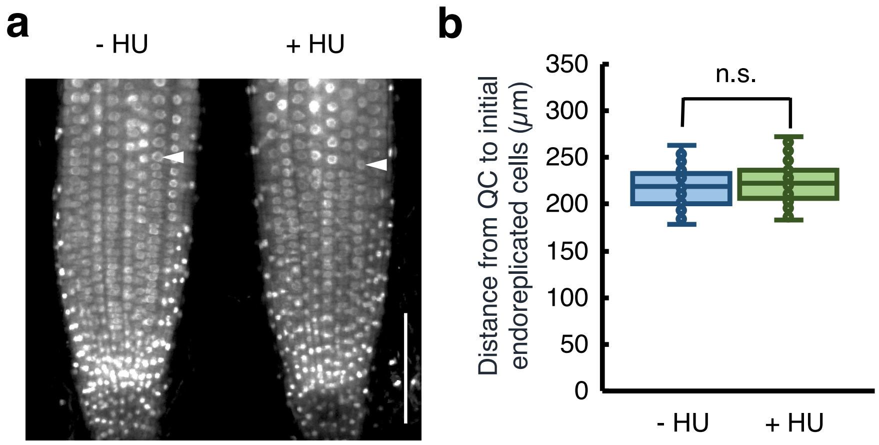

Fig. S2 Hydroxyurea treatment does not promote the early onset of endoreplication in root tips (**a**) Representative images of DAPI-stained nuclei of roots. Five-day-old WT were transferred onto MS plates supplemented with (+ HU) or without (- HU) 2 mM hydroxyurea and grown for 24 h. Roots were stained with DAPI and observed using confocal laser scanning microscope. Arrowheads indicate the first endoreplicated nuclei in the epidermal hair cell layer. Bar = 100 μm. (**b**) The distance from the quiescent center (QC) to the first endoreplicated nucleus in individual epidermal hair cell layer (n > 20). No significant difference (n.s.) from the control was determined (Student's t-test).

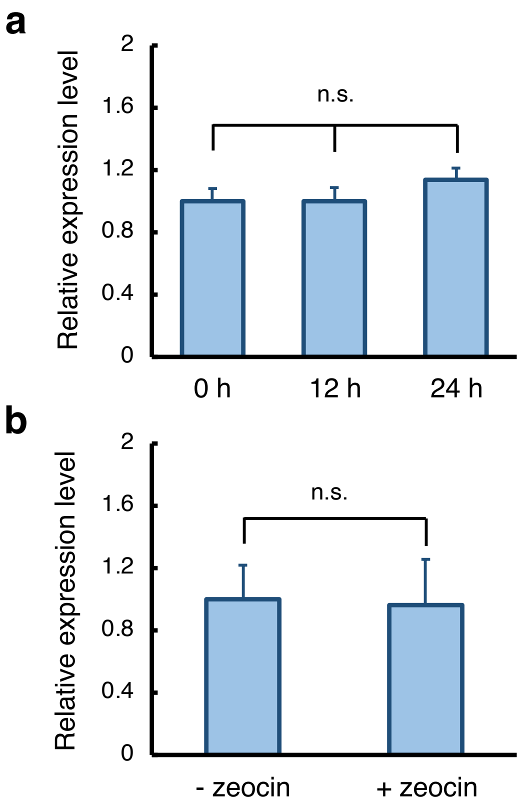

Fig. S3 Transcript level of *CCS52A2* under zeocin treatment. (**a**) Five-day-old WT and *sog1-101* seedlings were transferred onto MS plates supplemented with 8 µM zeocin and grown for 0, 12, and 24 h. Total RNA was extracted from roots and subjected to qRT-PCR. Transcript level of *CCS52A2* was normalized to that of *ACTIN2*, and are indicated as relative values, with the value at 0 h set to 1. Data are presented as mean ± SD calculated from three biological and technical replicates. No significant difference (n.s.) from the 0 h control was determined (Student's *t*-test). (**b**) Five-day-old *ccs52a1-1* seedlings were transferred onto MS plates supplemented with (+ zeocin) or without (- zeocin) 8 µM zeocin and grown for 24 h. Transcript level of *CCS52A2* was normalized to that of *ACTIN2*, and are indicated as relative values, with that for the control (- zeocin) set to 1. Data are presented as mean ± SD calculated from three biological and technical replicates. No significant difference (n.s.) from the control was determined (Student's *t*-test).

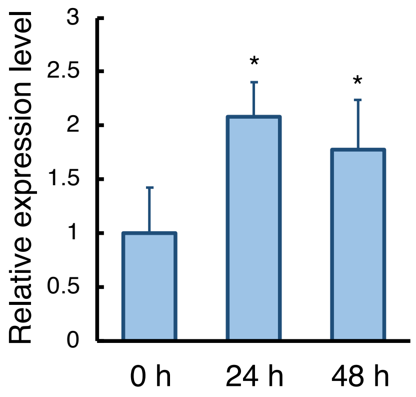

Fig. S4 Transcript level of *CCS52A1* in the *log7* mutant. Five-day-old *log7-1* seedlings were transferred onto MS plates supplemented with (+ zeocin) or without (- zeocin) 8 μM zeocin and grown for 0, 24 and 48h. Transcript level of *CCS52A1* was normalized to that of *ACTIN2*, and are indicated as relative values, with that for the control (- zeocin) set to 1. Data are presented as mean ± SD calculated from three biological and technical replicates. Significant differences from the control were determined by Student's *t*-test: $*P < 0.05$.
